# The Bright Elusive Butterfly of Value in Health Technology Development: Comment on "Providing Value to New Health Technology: The Early Contribution of Entrepreneurs, Investors, and Regulatory Agencies"

**DOI:** 10.15171/ijhpm.2017.65

**Published:** 2017-05-29

**Authors:** Trisha Greenhalgh, Nick Fahy, Sara Shaw

**Affiliations:** Nuffield Department of Primary Care Health Sciences, University of Oxford, Oxford, UK.

**Keywords:** Innovation Policy, Health Policy, Health Technology Assessment, Technology-Based Ventures, Health Research Systems

## Abstract

The current system of health technology development is characterised by multiple misalignments. The "supply" side (innovation policy-makers, entrepreneurs, investors) and the "demand" side (health policy-makers, regulators, health technology assessment, purchasers) operate under different – and conflicting – logics. The system is less a "pathway" than an unstable ecosystem of multiple interacting sub-systems. "Value" means different things to each of the numerous actors involved. Supply-side dynamics are built on fictions; regulatory checks and balances are designed to assure quality, safety and efficacy, not to ensure that technologies entering the market are either desirable or cost-effective. Assessment of comparative and cost-effectiveness usually comes too late in the process to shape an innovation’s development.

We offer no simple solutions to these problems, but in the spirit of commencing a much-needed public debate, we suggest some tentative ways forward. First, universities and public research funders should play a more proactive role in shaping the system. Second, the role of industry in forging long-term strategic partnerships for public benefit should be acknowledged (though not uncritically). Third, models of "responsible innovation" and public input to research priority-setting should be explored. Finally, the evidence base on how best to govern inter-sectoral health research partnerships should be developed and applied.

In a recent overview,^1^ Pascale Lehoux and her team synthesise and extend a series of papers on different aspects of a large, rigorously conducted and richly theorised qualitative case study of the fortunes of five health technology start-ups, based on five years’ fieldwork in Quebec, Canada (2008-2013).^2-4^ They also draw on their previous studies on other health technologies and stakeholders^5-8^; on theories of institutions,^9^ business modelling,^10^ co-construction of technology^11^ and sociology of expectations^12^; and on a systematic review of early health technology assessment.^13^

Their key messages, which are all the more poignant for being understated, are threefold:

1. The current system of health technology development is full of misalignments.
2. Nobody – or almost nobody – fully understands what is going on.
3. There is much avoidable waste.

Let us take some actors in the health technology development drama – whose plot turns on the societal tension between private gain and public good – and follow them as they as they strut and fret their hour upon the stage.

The policy-maker who works for the government’s innovation department knows that she is operating in a fast-moving, capital-intensive knowledge economy. She is tasked with supporting technology start-ups and engaging suppliers in a way that promotes growth, creates highly-qualified jobs and makes the country more internationally competitive in technology.^14^ She adjusts procurement processes and tax incentives accordingly and designates a pot of money for “innovation,” prioritising small and medium-sized enterprises (which she views as a rich source of creative ideas).

The clinical entrepreneur has an idea for a technology that will (he imagines) improve an aspect of clinical management (screening, diagnosis, treatment, monitoring, self-care etc). He bids successfully for government seed funding and generates a prototype, perhaps in partnership with a bioengineer. What they lack in business expertise they make up for in enthusiasm and confidence. Their ambition is to commercialise the technology, improving an aspect of care for patients while also making them some money. But they have only a hazy idea of how to bring their product to market.

Our heroes (the entrepreneur-bioengineer partnership) raise some small-scale development funding (perhaps by investing in the venture themselves). They undertake initial proof-of-concept studies and secure a patent to protect their intellectual property. Believing (wrongly) that they are now nearing the end of the technology development process, they set up a company of which they are both co-directors. But they quickly discover that the path to “health” (for patients) and “wealth” (for themselves) is neither straight nor smooth. Their technology cannot be used on patients until it has met regulatory approvals; regulatory approvals require stringent tests of quality, safety and efficacy; those tests require substantial money; and money up-front requires a business plan and a plausible promise of return on investment. Our entrepreneur begins to learn the language of venture capital; he crafts a (fanciful, simplistic) narrative linking the clinical need (problem) with his technological innovation (solution) and sends it out to prospective investors.

The investor in this drama is used to dealing with enthusiasts who do not understand financial markets or regulatory requirements. She knows that healthcare technologies often fail to fulfil their early promises but occasionally prove to be genuine (and profitable) breakthroughs. She is attracted to novel ideas and skilled in diversifying her portfolios so as to distribute risks. But her shareholders expect a good return within a few years. She will not put money into a venture solely on the basis of the latent (that is, tentative) value proposition expounded by the entrepreneur. She looks for objective data to estimate the size of the potential market and pace of development, and gauges what level of interest other investors are showing. She reads the business and financial media carefully.

The business analyst (journalist, blogger etc) writes creatively for the investment market. Using a genre of writing that is unashamedly speculative and which some would describe as hyperbolic, he crafts a narrative that reframes the problem-solution dyad in quantitative financial terms. This narrative emphasises four things: risk (if the condition remains untreated, there will be x amount of suffering, expense, litigation etc), efficiency (routine use of the technology will save y bed days, clinic visits, malpractice suits etc), profit (once the technology has been widely adopted, an annual return of $z is predicted) and trustworthiness (the company’s incoming chief executive has a strong track record of generating high returns in a related business). The purpose of this narrative is not to provide a detailed exposition of clinical need or an accurate estimate of benefit and harm but to engender sufficient interest (nay, excitement) from investors to attract an initial tranche of venture capital. Because investors’ unit of analysis is not the individual venture but the diversified portfolio, it helps to be as sensational as possible. Indeed, this is necessary because it helps to ensure that money flows in the direction of risky ventures, a tiny fraction of which will be highly successful.

On the basis of one such necessary fiction, our investor cautiously decides to finance our heroes’ venture. Before putting money on the table, she takes steps to “de-risk” it to increase the chances that it will generate an early return. The technology must be capable of being mass-produced and commercialised (that is, sold on as a viable business proposition). Nice-to-have but expensive-to-produce features must be pared back because they interfere with this goal. Otherwise, no deal. Our heroes are disappointed (they created all the bells and whistles and can justify their value, if not in financial terms), but they need the money so must accept a compromise. Lawyers are brought in to draw up contracts.

Once a deal has been struck, the investor begins to coach and nurture our entrepreneur and his board of directors. They must learn to focus clearly on the point in the not-too-distant future when the investment can be liquidated (eg, sold as shares) and its [financial] value realised. To that end, they must produce a revenue model (an account of how, when and to whom sales will occur), grow a value network (relationships with suppliers, distribution channels and competitors) and tighten every step in the value chain. Milestones, especially regulatory approval (which will enhance the venture’s economic and clinical value in the eyes of investors) must be explicit and measurable. If they slip, our hard-nosed investor will intervene actively to modify the business strategy and bring the venture back on [financial] course. Subsequent tranches of funding will be contingent on complying with such intervention. Thus, the idea becomes progressively more viable as a business venture – but, not uncommonly, loses features that cannot be quickly monetised.

There are multiple gatekeepers (sometimes known as regulators) in this story. One works for an institution that grants licences for the use of health technologies; his concern is the technology’s quality, safety and the basic “does it work?” efficacy question. Investors will ensure that early health technology appraisals are explicitly designed to provide precisely the information this regulator needs (no more, no less) to award the licence. Another gatekeeper works for a financial institution; her concern is the auditability of businesses. The investor will guide the venture to put in place whatever the financial gatekeeper needs to grant market clearance.

The story thus far has articulated the supply side of the equation. Meanwhile, the healthcare policy-maker (who works in a different government department from the innovation policy-maker) and/or local purchaser are asking demand-side questions that reflect the (very different) priorities and values of health technology assessment (incidentally, we dispute Lehoux and colleagues’ use of the word “intrinsic” to describe these demand-side values: there are no *a priori* values). For which denominator populations will this technology bring benefit? What is the number needed to treat and number needed to harm for each sub-population (with confidence intervals please)? What alternative approaches (including doing nothing) are there, and how do their benefit-harm ratios compare? How affordable is this option given the overall health budget and opportunity costs? The evidence generated for regulatory approval is unlikely to address these questions unless they were anticipated and built in from the start. A new round of studies is commissioned (typically by a different agency), this time focusing on comparative and cost effectiveness.

In this penultimate scene, let us introduce our final character: the patient. Far from being the star of the show, he has only a cameo (and, usually, non-speaking) part in the drama. He may have featured fleetingly in earlier acts as an anonymised “subject” in research trials and thence as a dot on a graph in the appendix to a licence application. But what does he value? In what way is he ill (or at risk of illness) and what trade-offs is he prepared to make to reduce his actual or potential suffering? Taking account of those trade-offs, to what extent does he consider the new technology desirable? Has anybody asked him – and even if they did, were his views incorporated in the series of non-linear decisions that followed? Yet depending on how supply-side and demand-side logics play out, his condition may soon come to be investigated or treated – and in some cases, defined and even brought into being – using the new technology.

The best-case denouement of this drama is that despite the inherent malalignments in the system, a useful innovation still emerges, gains regulatory approval, gets taken up in practice to the benefit of patients and generates a profit for its developer and investors. But sometimes, because supply-side dynamics tend to distort and “freeze” the value of a health technology before the demand side gets a look-in, nobody gains. The patient does not gain (and may lose out) because the outcomes he values – and the trade-offs he considers worthwhile – never influenced the design or modification of the technology. The entrepreneur and bioengineer do not gain because the investors (and their lawyers) drove such a hard bargain that they will make little if any money when the venture is liquidated. Shareholders do not gain because despite gaining regulatory approval the venture never becomes viable (for example, because clinicians resist the new technology^15^ and/or the patient population that stands to benefit from it is too small to recoup production costs). The economy does not gain because the promise of highly-skilled jobs and greater international competitiveness is never realised. The healthcare system does not gain because nothing gets produced that is better or cheaper than the existing care model for the target condition.

*Curtain.*

Lehoux and colleagues’ account of health technology development resonates with previously published critiques of “irresponsible innovation” characterised by technology push, neglect of ethical principles, policy pull and lack of precautionary measures.^16-19^ We were, arguably, misled when governments wove together the terms “innovation,” “health” and “wealth” and implied that pursuit of the first would generate, inevitably and in parallel, the second two^20-22^; empirical evidence suggests it rarely does.^23-27^

We agree that current incentive and regulatory mechanisms are not supporting or rewarding the public goods that are needed in the health system. But we believe there is evidence that the research system is already playing a more significant role in the supply side of the equation than Lehoux and colleagues imply. If incentives were better aligned, universities could potentially become “lead actors” that could powerfully shape supply-side dynamics.^28^

That said, there are also potential further misalignments between the research system and the health system that Lehoux et al have not explored, but which may be evident in their extensive dataset. For example, many academics consider that digital technologies should be tested using “gold standard” randomised trial methods, characterized by narrow research questions, pre-specified user groups and outcomes, procedural rigidity and efforts to “control out” the effects of material, social, and political context.^29^ Even if efficacy is demonstrated through such trials, the technology is likely to be obsolete by the time the findings are published and the political and purchasing realities that could stymie its adoption and spread in practice will have been overlooked – as illustrated by the UK’s multi-million pound ‘Whole Systems Demonstrator’ mega-trial of telehealth.^30^

Another apparent omission from Lehoux and colleagues’ empirical dataset is any direct capture of the patient voice, though they have previously demonstrated the importance of lay input in establishing the desirability (or not) of health technologies.^5,7^ Formal priority-setting partnerships such as the UK’s James Lind Alliance^31,32^ or public deliberation platforms^18^ are two contrasting approaches to gaining lay input to the research agenda, thereby potentially reducing waste.^33^ However, the conventional separation of “innovation” budgets from “research” budgets means that such approaches may have limited influence on technology start-ups. Our own ethnographic study of the priorities of older people for assisted living technologies found low levels of adoption and use (and, not uncommonly, active concealment or sabotage) of “innovative” technologies along with creative efforts by patients and their carers to build their own solutions by repurposing familiar technologies available within the home.^34^

There is emerging (and reassuring) evidence of a radical change in the strategy taken by large technology companies to developing implementable and scalable technologies for healthcare. In our early fieldwork on electronic patient records and assisted living technologies (2005-2013), we observed a number of instances of aggressive marketing of off-the-shelf technologies to UK purchasers by leading suppliers.^34,35^ Many such technologies were sold through block contracts but only a fraction was ever used as they proved unfit for purpose and had not included a co-design element. Some companies developed a reputation for “shark” marketing tactics oriented towards gaining a short-term sale in the interest of maximizing profit. In the past three years, however, we have documented an increasing willingness by such companies to engage in long-term strategic partnerships with health and social care organisations, promote open standards, data exchange and interoperability in ways that facilitate collaboration across suppliers and increase potential for widespread adoption, undertake ethnographic studies and co-design projects, and hire clinical staff with extensive patient-facing experience (unpublished data from research interviews with executives from Microsoft, Tunstall and Philips).

Various efforts have been made at policy level in different countries to better align the early research and development process with health needs. For example, the European Commission has developed special systems for funding, licensing and evaluating medicines for rare diseases, as well as advance purchase commitments for neglected diseases.^36^ But the exceptional nature of such arrangements highlights the challenges of trying to manage such an inherently complex system. In an era of increasingly complex inter-sectoral health research systems,^37^ there is an emerging evidence base (though no simple solutions) on how to govern and incentivise these “managed networks” to help align disparate goals and maximise value for all stakeholders.^38,39^ Some countries have an independent “council for health” (for example, De Gezondheidsraad in the Netherlands) that is independent of government and operates in a neutral space to help facilitate the work of the multiple stakeholders and the interactions between them in a way that is clearly focused on maximising health gain.

In conclusion, we concur with Lehoux et al that health technology development is not (and never will be) a smooth pipeline. We commend them for proposing an initial model for illustrating how the value proposition of a new technology is affected by both supply-side and demand-side influences (which are often poorly aligned). In this commentary, we have highlighted some additional influences (both positive and negative) that might need to be factored into this model. We have re-drawn Lehoux and colleagues’ model to embrace these additional influences and indicated where more strategically-directed research (of various kinds) might help to reduce misalignments and waste in the system (Figure). This model is by no means definitive and we look forward to further discussion and debate.

**Figure:**
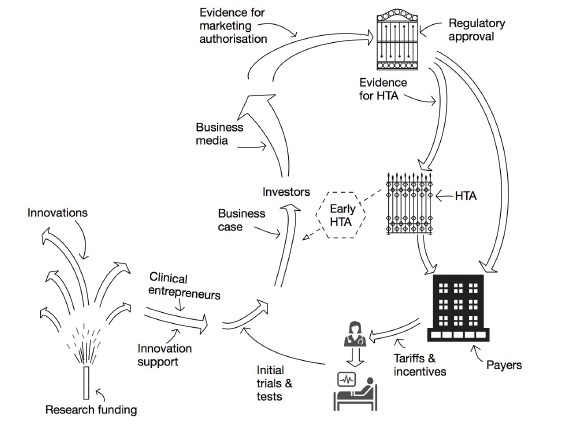
Key Influences on the Value Proposition of a New Technology. Adapted from Lehoux et al.^1^

## Ethical issues

Not applicable.

## Competing interests

Authors declare that they have no competing interests.

## Authors’ contributions

TG wrote the initial draft of the paper, which was amended by input from SS and NF. NF drew the figure.

## Funding

The authors received no fee for this commentary, which was independently peer reviewed. TG, SS, and NF’s salaries are funded by the National Institute for Health Research Biomedical Research Centre, Oxford, UK (grant NIHR-BRC-1215-20008) and by additional grants from the National Institute for Health Research (VOCAL study, NIHR13/59/26). The paper was drafted when TG was on a writing retreat at the Bellagio Center, Bellagio, Italy, funded by the Rockefeller Foundation.
